# Cell populations can use aneuploidy to survive telomerase insufficiency

**DOI:** 10.1038/ncomms9664

**Published:** 2015-10-22

**Authors:** Caroline Millet, Darya Ausiannikava, Thierry Le Bihan, Sander Granneman, Svetlana Makovets

**Affiliations:** 1 Institute of Cell Biology, School of Biological Sciences, University of Edinburgh, Edinburgh EH9 3FF, UK; 2 Centre for Synthetic and Systems Biology (SynthSys), School of Biological Sciences, University of Edinburgh, Edinburgh EH9 3JD, UK

## Abstract

Telomerase maintains ends of eukaryotic chromosomes, telomeres. Telomerase loss results in replicative senescence and a switch to recombination-dependent telomere maintenance. Telomerase insufficiency in humans leads to telomere syndromes associated with premature ageing and cancer predisposition. Here we use yeast to show that the survival of telomerase insufficiency differs from the survival of telomerase loss and occurs through aneuploidy. In yeast grown at elevated temperatures, telomerase activity becomes limiting: haploid cell populations senesce and generate aneuploid survivors—near diploids monosomic for chromosome VIII. This aneuploidy results in increased levels of the telomerase components TLC1, Est1 and Est3, and is accompanied by decreased abundance of ribosomal proteins. We propose that aneuploidy suppresses telomerase insufficiency through redistribution of cellular resources away from ribosome synthesis towards production of telomerase components and other non-ribosomal proteins. The aneuploidy-induced re-balance of the proteome via modulation of ribosome biogenesis may be a general adaptive response to overcome functional insufficiencies.

## Abstract (web summary)

The loss of telomeres is a catastrophic event and eukaryotes have evolved multiple strategies to overcome this. Here the authors show that *Saccharomyces cerevisiae* can generate aneuploid survivors that upregulate telomerase to overcome telomere loss.

## Graphical abstract

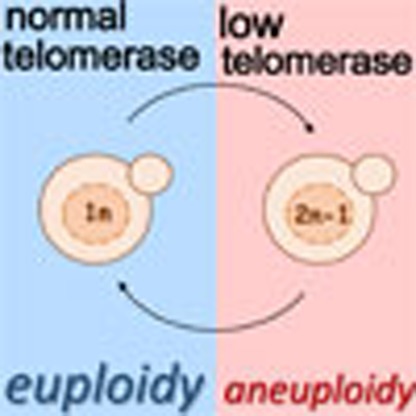

Telomeres are ends of linear eukaryotic chromosomes, which in most organisms are maintained by the enzyme telomerase. Telomerase extends telomeres, thereby compensating for telomere shortening caused by DNA replication^1^. In humans, the expression of the telomerase catalytic subunit hTERT is strongly downregulated in somatic cells^2^. This repression may act as a barrier to oncogenesis as precancerous cells must overcome the problem of telomere maintenance to proliferate indefinitely. Only 10–15% of cancers are telomerase negative and use recombination to maintain their telomeres, while the majority reactivate enough telomerase to keep telomeres short, but stable^3^. Recent discoveries linked telomerase reactivation in some tumours to mutations in the *hTERT* promoter^4^. However, such mutation were not found in other telomerase-positive cancer cells^5^ and the mechanisms of telomerase reactivation for these cancers remain unknown.

In dividing yeast, telomerase loss results in gradual telomere shortening, activation of the DNA damage checkpoint and massive cell death followed by appearance of infrequent survivors, which maintain telomeres via recombination^6^,^7^,^8^,^9^,^10^,^11^,^12^,^13^.

Laboratory strains of yeast *Saccharomyces cerevisiae* are typically propagated at 30 °C and possess 300–350 bp of telomeric repeats (TG_1-3_)_*n*_ (ref. 14), but at higher temperatures the telomere length equilibrium is shorter^15^. When haploid, *S. cerevisiae* possess 16 chromosomes and 32 telomeres maintained by telomerase. Telomerase is a reverse transcriptase^16^ that uses an integral RNA component, TLC1 in yeast, as a template to extend the 3′ end of a telomere^13^. Est2 is the yeast telomerase catalytic protein subunit while Est1 interacts with TLC1 (ref. 17) and the telomere-binding protein Cdc13 (ref. 18) to recruit telomerase to telomeres. Est3 interacts directly with Est1 and Est2 and stimulates Est2 catalytic activity^19^. All the telomerase components are present *in vivo* in low abundance, with TLC1 being the limiting factor^20^. Although *TLC1* is haploinsufficient in diploids, the slightly shorter telomeres in *TLC1/tlc1Δ* cells at 30 °C are stable and do not limit cell proliferation^20^.

Loss of any of the telomerase components does not cause immediate cell death. DNA replication in telomerase-negative cells leads to gradual telomere shortening until one or more critically short telomeres, which are recognized as double-strand breaks, trigger the DNA damage checkpoint and result in cell cycle arrest and loss of viability^6^,^7^,^8^,^9^,^10^,^11^,^12^,^13^. This viability crisis is followed by the generation of rare survivors that maintain their telomeres via recombination involving either sub-telomeric Y′ repeats (type I survivors) or telomeric (TG_1-3_)_*n*_ repeats (type II survivors)^6^,^7^,^21^.

In contrast to complete telomerase loss, telomerase insufficiency, when cell proliferation is limited by low amount of telomerase, is more closely relevant to human telomere syndromes^22^ and cancer. Below we show that, unlike the use of recombination to overcome telomerase deletion, when faced with telomerase insufficiency yeast cells may use chromosome-specific aneuploidy to upregulate telomerase and maintain short but stable telomeres. This aneuploidy leads to downregulation of ribosomal proteins accompanied by increased abundance of various non-ribosomal proteins and RNA, which include the telomerase protein components Est1, Est3 and telomerase RNA TLC1. Our results provide strong evidence that in yeast, survival of telomerase insufficiency differs from overcoming telomerase loss and can occur through aneuploidy-dependent upregulation of telomerase. Since cancer cells are highly aneuploid^23^ and many increase telomerase activity to propagate indefinitely^2^, aneuploidy may be an evolutionarily conserved means to elevate telomerase activity and maintain proliferative capacity.

## Results

### Telomerase insufficiency in yeast grown at 38.5 °C

**Figure 1:**
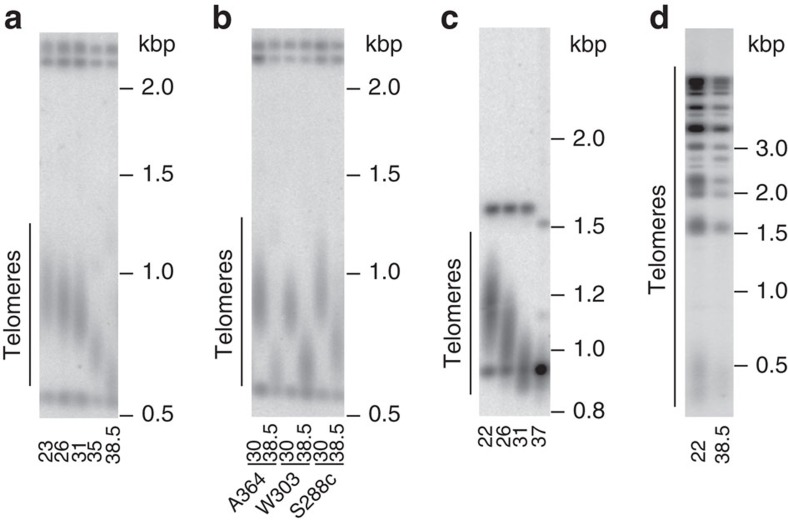
Telomere length equilibrium is temperature dependent in budding yeast *S. cerevisiae* and fission yeast *S. pombe* but not in algae *C. vulgaris*. (**a**–**d**) Southern blot analysis of telomere length equilibrium after cells were grown for four passages (∼80 generations) at the temperatures shown at the bottom of the corresponding lanes on blots. DNA size marker is shown on the right of each blot. (**a**) *S. cerevisiae* A364a Y′ telomeres. (**b**) Y′ telomeres of the three *S. cerevisiae* strain backgrounds, A364a, W303 and S288c, commonly used in research. (**c**) *S. pombe* telomeres. (**d**) *C. vulgaris* telomeres.

Consistent with the previously published data^15^, telomeres in the budding yeast *S. cerevisiae* equilibrated at a shorter average length when cells were propagated for 80 generations (four passages, each passage equals ∼20 cell doublings) at higher temperatures (Fig. 1a,b). This re-equilibration was also characteristic of the fission yeast *Schizosaccharomyces pombe* when propagated at higher temperatures, but it was not characteristic of the algae *Chlorella vulgaris* (Fig. 1c,d).

**Figure 2:**
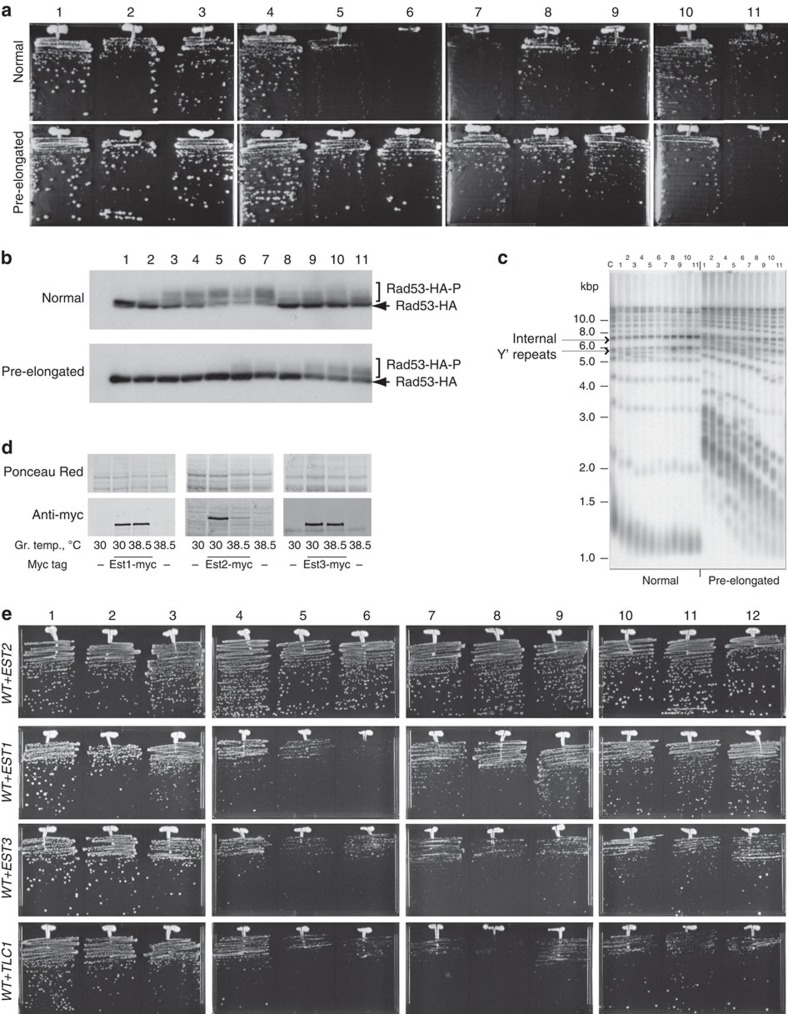
Viability crisis during yeast cell growth at 38.5 °C is caused by telomerase insufficiency. (**a**) Plate images of serial passaging at 38.5 °C of cells after their telomeres were equilibrated at 30 °C either at the wild-type length (‘normal’, upper panel) or much longer (‘pre-elongated’, lower panel). Here and in further figures, the passage number is indicated above images. (**b**) DNA damage signalling activation during the passaging in **a**, as monitored by Rad53 phosphorylation (Rad53-HA-P). (**c**) Telomere length dynamics during the passaging in **a**, as assayed by Southern blotting. A control sample (C) from wild-type (WT) strain grown at 30 °C is in the left most lane. (**d**) The effect of elevated growth temperature on the steady-state levels of telomerase subunits Est1, Est2 and Est3, analysed by western blotting (bottom panels). All the analysed proteins contained 13myc tags at the C termini. Equal amounts of total cell protein were loaded in each lane (see upper panels for Ponceau Red staining of total protein on blotting membranes). (**e**) A second copy of *EST2* but not *EST1*, *EST3* or *TLC1* suppresses telomerase insufficiency at 38.5 °C. Serial passaging of haploid cells with an additional copy of *EST1*, *EST2*, *EST3* or *TLC1* (as indicated on the left) integrated into a genomic locus of a WT strain.

Propagation of *S. cerevisiae* at 38.5 °C for longer than 80 generations resulted in a viability crisis at passages 3–7 (Fig. 2a, upper panel) followed by the generation of ‘temperature survivors’ (Fig. 2a, upper, passages 8–11). The cell growth was accompanied by activation of a DNA damage checkpoint as evident from the phosphorylation of Rad53, the key kinase in the pathway (Fig. 2b, upper panel). The maximum level of Rad53 phosphorylation correlated with the peak of cell viability crisis (Fig. 2b, upper panel, passages 5–7). Analysis of telomere length revealed that telomeres continued to shorten during earlier passages, after which the telomeres remained short and stable (Fig. 2c, left panel and Supplementary Fig. 1).

To test whether the cell viability crisis was elicited by critically short telomeres, we pre-elongated telomeres by temporarily expressing the *CDC13*–*EST2* gene fusion^24^ and then selecting for its loss before cells were propagated at 38.5 °C (Fig. 2c, right panel and Supplementary Fig. 1). We reasoned that cells with pre-elongated telomeres would exhibit a delay in the viability crisis as it would take longer for their telomeres to become critically short. Telomere pre-elongation delayed both the viability crisis and Rad53 phosphorylation until streak 11 (Fig. 2a, lower panel and Fig. 2b, lower panel). Thus, telomere maintenance is the growth-limiting factor at 38.5 °C. Analysis of a larger number of ‘temperature survivors’ showed that the majority possessed short and stable telomeres (Supplementary Fig. 2a), which is a phenotype distinct from type I and II telomerase-negative survivors (see Supplementary Fig. 2b for a comparison). In a control experiment, deletion of either *TLC1* or *EST2* at 38.5 °C led to the formation of type I and type II survivors (Supplementary Fig. 2b), indicating that survival of telomerase loss was not influenced by the elevated temperature and that recombination-dependent telomere maintenance can operate at 38.5 °C.

The shorter telomeres at higher growth temperature could result from a lower rate of telomere extension, a higher rate of telomere erosion (due to recombination or a nuclease-dependent DNA degradation at telomeres) or both. To distinguish between these possibilities, we compared the rates of telomere shortening at 26 and 38.5 °C in cells lacking telomerase (*est2Δ* or *tlc1Δ*), exonuclease Exo1 (*exo1Δ*) or homologous recombination (*rad52Δ*). In the wild-type control, the effect of temperature on telomere length was readily observed after ∼20 generations, became more pronounced on ∼40 generations and was not affected by either *exo1Δ* or *rad52Δ* (Supplementary Fig. 3a, left and middle panels). Furthermore, both *exo1Δ* and *rad52Δ* mutants resembled wild-type cells by undergoing a viability crisis at 38.5 °C (Supplementary Fig. 3b) suggesting that neither Exo1-dependent DNA degradation nor recombination at telomeres was responsible for the viability crisis. In contrast, the growth temperature did not affect telomere length in the telomerase mutants, as their telomeres shortened at the same rate at 26 and 38.5 °C (Supplementary Fig. 3a, right panel). Because the telomere erosion was temperature independent, we inferred that the growth temperature affected telomerase-dependent telomere lengthening. The effect of growth temperature on the telomerase complex components Est1, Est2 and Est3 was analysed and the steady-state level of the telomerase catalytic subunit Est2 was markedly reduced at 38.5 °C (Fig. 2d). Consistent with these observations, the cell viability crisis at 38.5 °C was suppressed when a second copy of *EST2* but not *EST1*, *EST3* or *TLC1* was integrated into the yeast genome (Fig. 2e). Therefore, the viability crisis at 38.5 °C was caused by the insufficiency of telomerase activity, most likely due to limiting Est2, and we named the cells that survived the crisis telomerase insufficiency survivors (TI survivors).

### CHR VIII monosomy suppresses telomerase insufficiency

**Figure 3:**
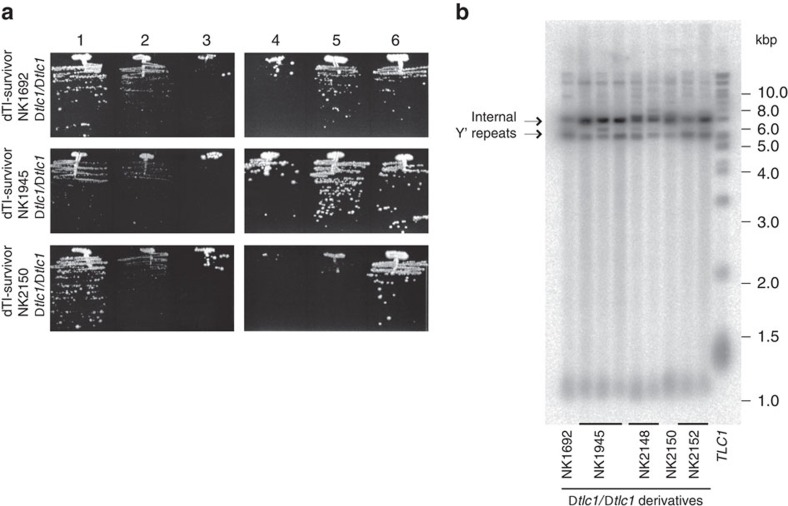
dTI survivors require telomerase for their viability. (**a**) Plate images of serial passaging at 30 °C of three dTI survivors after telomerase inactivation with deletion of both copies of *TLC1*. (**b**) Telomere analysis of *tlc1Δ/tlc1Δ* derivatives of dTI survivors from the passage 6 shown in **a** (not all the clones are shown in passaging). The right most lane shows telomeres from a control sample, wild-type cells grown at 30 °C. Notice that *tlc1Δ/tlc1Δ* derivatives of all the dTI survivors formed type I survivors. Amplification of internal Y′ repeats often seen in TI survivors might be pre-disposing them to the type I pathway after telomerase loss.

We next analysed TI survivors generated from the propagation of 79 independent clones at 38.5 °C by fluorescence-activated cell sorting (FACS). To our surprise, 32 clones possessed a DNA content characteristic of diploid cells, but retained Mat-a phenotype (that is, they were not true diploids, which would express both *MATa* and *MATα*). To test if the diploid TI survivors (dTI survivors) maintained their telomeres via telomerase, *tlc1Δ/tlc1Δ* derivatives were generated. All the telomerase-deficient dTI survivors exhibited a viability crisis (Fig. 3a) followed by the formation of type I survivors (Fig. 3b). Therefore, the dTI survivors are different from the survivors generated in response to telomerase loss and rely on telomerase for telomere maintenance.

**Figure 4:**
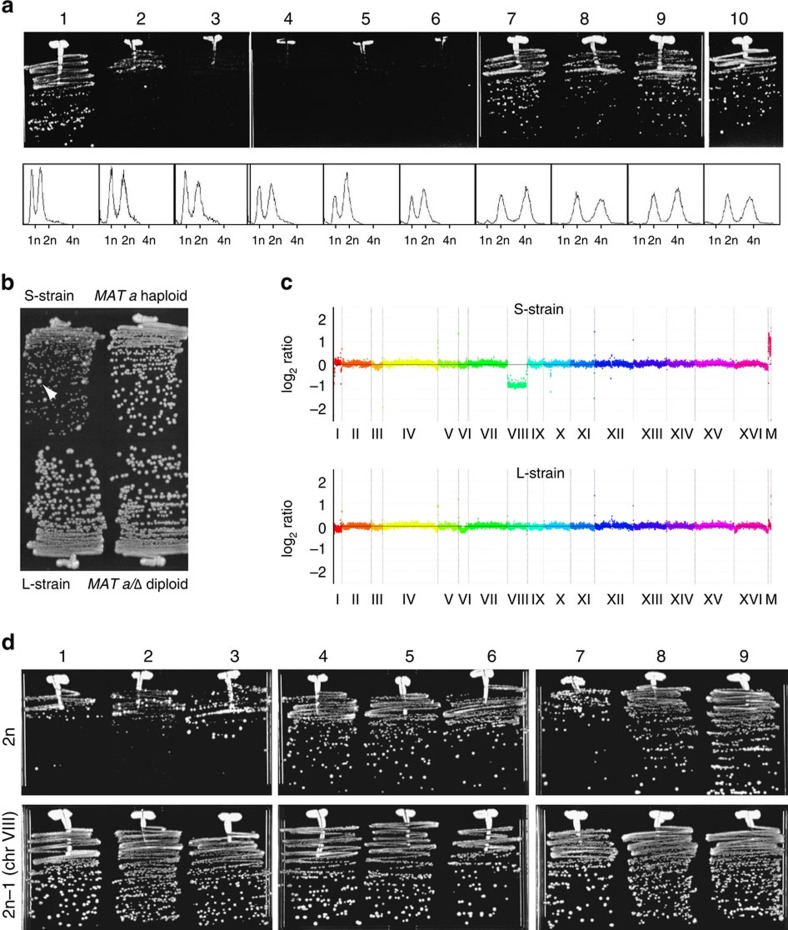
CHRVIII aneuploidy suppresses telomerase insufficiency. (**a**) Plate images of serial passaging of haploid yeast at 38.5 °C along with the FACS analysis of cells from each passage shown below the corresponding streak image. DNA content of 1n, 2n and 4n is indicated at the *x* axes of the FACS profiles. Notice that the switch from a haploid to a diploid state (from peaks 1n/2n to 2n/4n) coincides with the appearance of healthy growing survivors at passage 7. (**b**) At 30 °C, dTI survivors have small colony phenotype (S strains) but generate some faster growing colonies (indicated by white arrow), which stably inherit the growth phenotype (L strain). Control haploid and diploid strains are shown for comparison. (**c**) aCGH analysis of the S and L strains reveals CHRVIII aneuploidy and an increase in mtDNA (labelled M) in the S strain (Supplementary Fig. 4). (**d**) Plate images of serial passaging at 38.5 °C of *matΔ/MATα* fully diploid strain (2n, upper panel) and its CHRVIII aneuploid derivative (2n−1, lower panel).

Analysis of DNA content throughout the propagation period at 38.5 °C revealed a correlation between the timing of ploidy switch and survivor formation (for example, Fig. 4a, streak 7), suggesting that diploidization could be a means to adapt to telomerase insufficiency. When passaged at 30 °C, the dTI survivors showed slower growth (small colonies, S strains) in comparison to either haploid or diploid yeast, but often generated larger colonies (L strains), that at 30 °C maintained a growth rate similar to that of wild-type yeast (Fig. 4b). The DNA content of both L and S strains was ∼2n by FACS. To uncover the difference between the L and S strains we used array comparative genome hybridization (aCGH) on three L/S pairs. All the three S strains had one copy of chromosome VIII (CHRVIII) and an increased amount of mtDNA, while the L strains regained the second copy of CHRVIII and re-equilibrated their mtDNA to near wild-type copy number (Fig. 4c and Supplementary Fig. 4). Thus, the dTI survivors shared in common a ploidy transition from 1n to near 2n, with CHRVIII monosomy and an increased mtDNA copy number. The aCGH analysis was extended to 10 additional dTI survivors and all of them contained a single copy of CHRVIII and increased mtDNA. In contrast, 10 haploid TI survivors analysed by aCGH did not show any change in relative DNA content throughout the genome and were not analysed further.

The mtDNA increase observed in the 2n−1 dTI survivors was not required for adaptation to telomerase insufficiency as yeast lacking mtDNA (ρ^0^ cells) passaged at 38.5 °C formed diploid-like survivors with a single copy of CHRVIII (Supplementary Fig. 5). To test if the karyotype 2n−1 (CHRVIII) suppressed telomerase insufficiency in dTI survivors, a *matΔ::KAN /MATα* diploid strain and its derivative carrying only a single copy of CHRVIII were genetically engineered at 30 °C and propagated at 38.5 °C (Fig. 4d). The complete diploid strain exhibited a mild viability crisis at early passages (Fig. 4d) while the strain monosomic for CHRVIII exhibited no growth defect (Fig. 4d, bottom panel). These results demonstrate that transition to the aneuploidy 2n−1 (CHRVIII monosomy) was sufficient to suppress the growth crisis induced by telomerase insufficiency in haploid cells (Fig. 2a).

To assay CHRVIII stability and its dependence on telomere length at 38.5 °C we passaged colonies from a *matΔ::KAN /MATα* diploid and its *CDC13/cdc13::CDC13–EST1* derivative with longer telomeres^24^. In the *CDC13/CDC13* cells, CHRVIII was lost during earlier passages but the onset of aneuploidy was significantly delayed in the presence of the Cdc13–Est1 fusion protein (Supplementary Fig. 6). In contrast, both copies of chromosome III were retained by all clones (n=54). Thus, CHRVIII aneuploidy in response to telomerase insufficiency is chromosome specific and can be observed in diploid cells.

### How CHRVIII monosomy may suppress telomerase insufficiency

**Figure 5:**
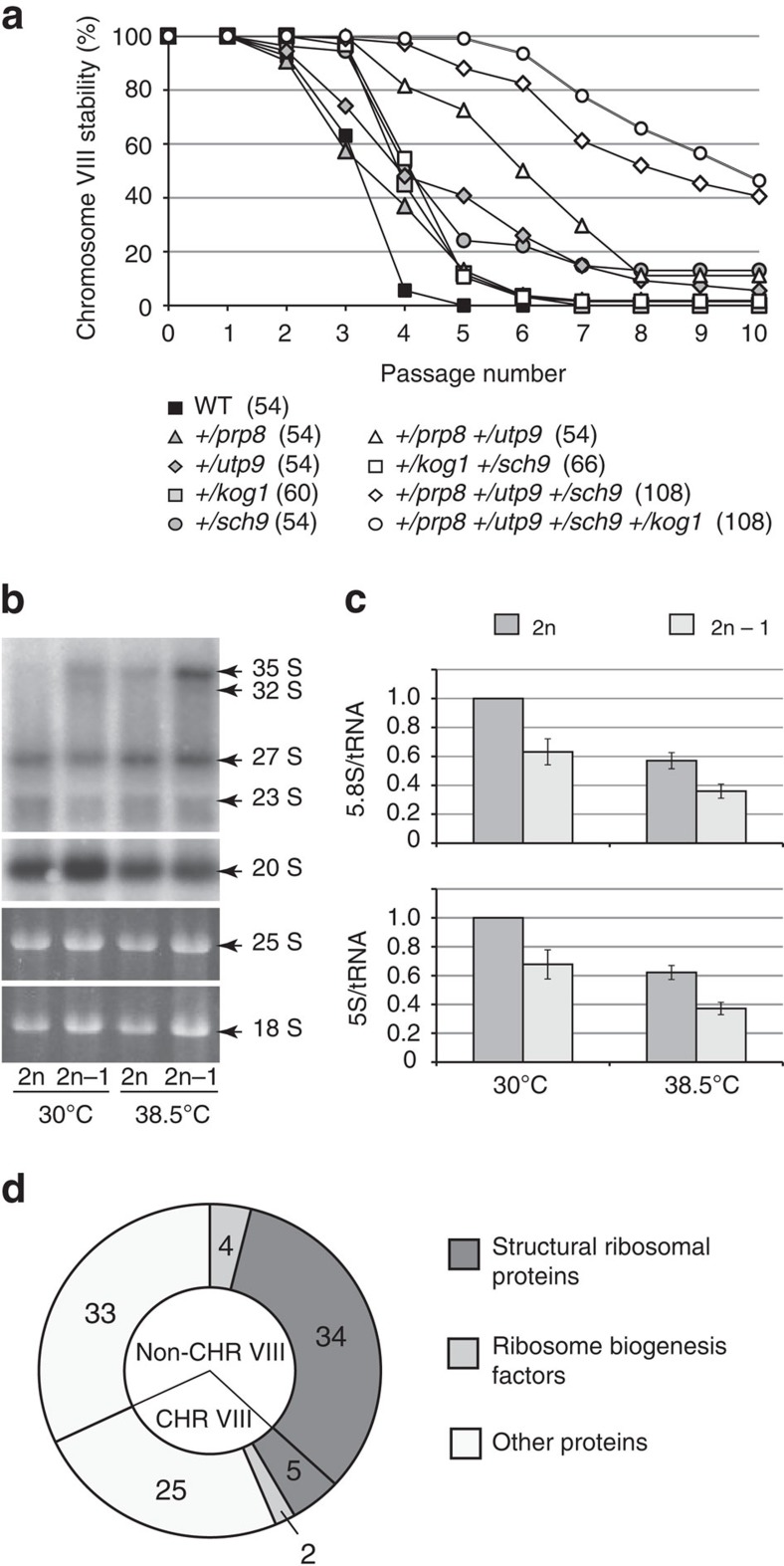
Downregulation of ribosome production in CHRVIII aneuploids might be responsible for the suppression of telomerase insufficiency. (**a**) Combined haploinsufficiencies in *UTP9*, *PRP8*, *SCH9* and *KOG1* delay CHRVIII loss. CHRVIII stability during serial passaging at 38.5 °C. Number of clones involved in the experiment is stated in brackets next to the corresponding genotype. (**b**) rRNA processing in diploid and aneuploid cells assayed by northern blotting (top two panels). The bottom two panels show total RNA resolved in agarose gel and stained with ethidium bromide before the blotting transfer. (**c**) CHRVIII aneuploidy results in lower steady-state levels of mature rRNA, 5.8S and 5S, when normalized to tRNA. All the relative amounts were normalized against diploids grown at 30 °C. Averages of three biological repeats for every experiment with error bars representing s.d.s are shown. (**d**) Abundance of 103 proteins was found reduced at least 1.5-fold in aneuploid cells, as detected by MS (*P*<0.05, analysis based on at least two different peptides for each protein, see Supplementary Data Set 1, group 1). Among them, 39 ribosomal proteins and 6 other proteins involved in ribosome biogenesis (Utp15, Rrp5, Snu13, Enp2, and CHRVIII-coded Nmd3 and Gar1). As expected, a large fraction of proteins in this group are encoded by genes on CHRVIII, which is reduced to a single copy in aneuploids. No ribosomal proteins with increased abundance in aneuploids were detected (Supplementary Data Set 1, group 2). See Supplementary Data Set 1 and Supplementary Tables 1–3 for the full data set and analysis of comparative MS.

To determine the mechanism by which CHRVIII aneuploidy enables the suppression of telomerase insufficiency, we searched for genes on CHRVIII, which, when reduced to a single copy in a diploid, would alleviate telomerase insufficiency and stabilize CHRVIII. Extensive analysis of CHRVIII stability in diploids carrying different deletions on one of the copies of CHRVIII identified four relevant genes: *PRP8*, *UTP9*, *KOG1* and *SCH9* (Supplementary Fig. 7). Heterozygosity of each of the genes did not elicit an observable impact on CHRVIII stability (Fig. 5a). However, when the mutations were combined in a quadruple heterozygous diploid of the genotype *+/prp8Δ +/utp9Δ +/kog1Δ +/sch9Δ*, the loss of CHRVIII was significantly delayed (Fig. 5a) although these mutations had only a moderate effect on telomere length at 30 °C (Supplementary Fig. 8). Utp9 plays a role in pre-rRNA processing^25^. Kog1 and Sch9 are components of the TOR signalling pathway, which positively regulates the transcription of ribosomal protein (RP) genes^26^, whereas Prp8 is an essential splicing factor^27^. In budding yeast, introns are found mainly in RP genes. Therefore, combined haploinsufficiencies in *PRP8*, *UTP9*, *KOG1* and *SCH9* (and perhaps other, yet unidentified genes on CHRVIII) might lead to a reduction in both the expression of RP genes and ribosome synthesis in CHRVIII aneuploids. Because RPs constitute ∼80–85% of total cell protein, we hypothesized that downregulation of RP gene expression might significantly increase the translation of less abundant non-RP mRNAs, such as Est1/2/3, explaining the suppression of telomerase insufficiency in CHRVIII aneuploids.

**Figure 6:**
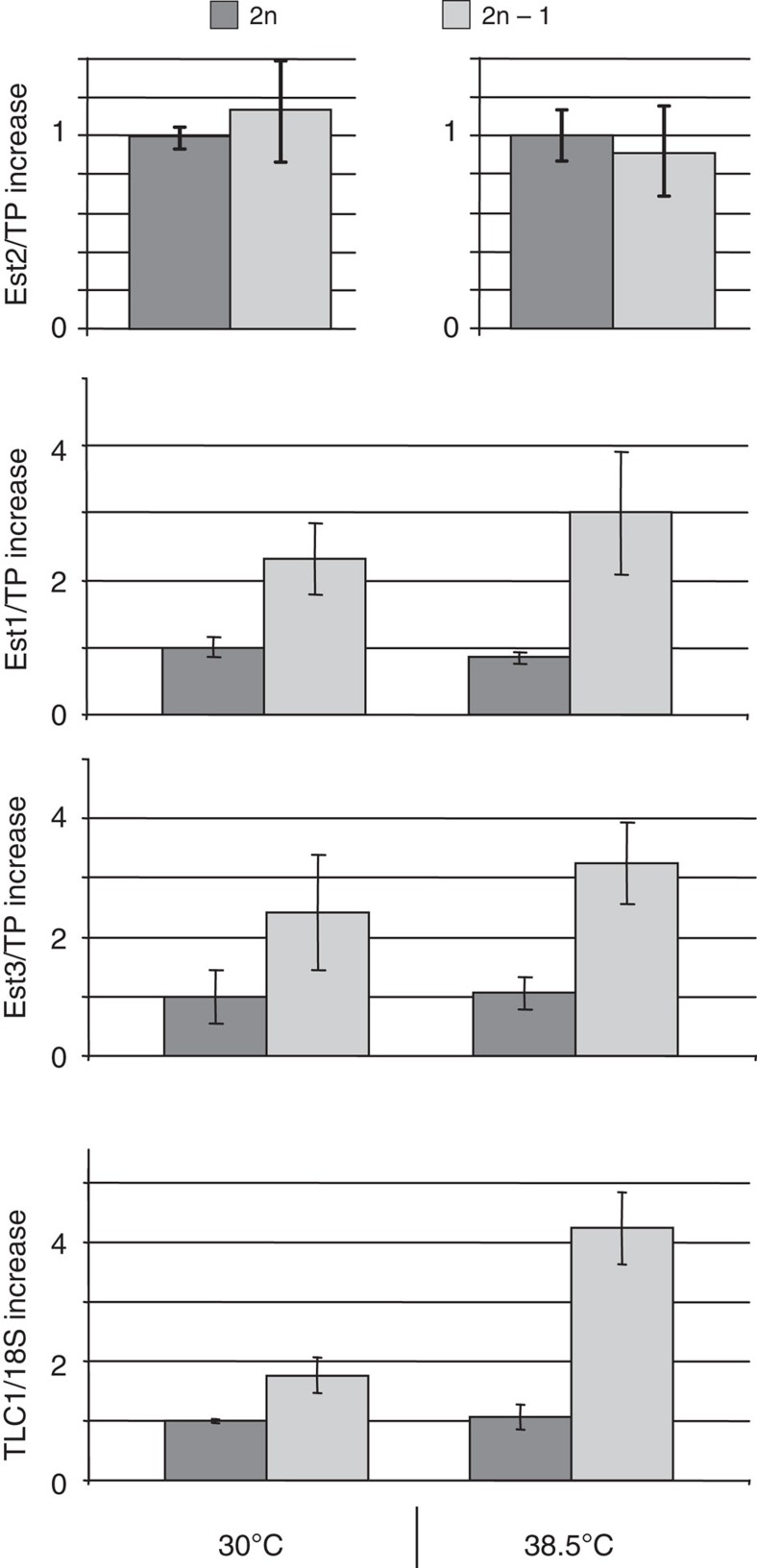
CHRVIII aneuploidy leads to increased levels of telomerase components. CHRVIII aneuploidy is accompanied by increased levels of TLC1 (normalized against 18S RNA), Est1 and Est3 (Est1/2/3 are normalized against total protein, TP). All the relative amounts are normalized against diploids grown at 30 °C, except for Est2 at 38.5 °C where the amount of Est2 was normalized against diploids grown at 38.5 °C. Comparative analyses of Est2 levels at different temperatures are shown on separate charts as the amount of Est2 at 38.5 °C was so low that immunoprecipitations of Est2-13myc from larger volume of cell lysates were required before the protein amounts could be compared by quantitative western blotting. Averages of three biological repeats for every experiment with error bars representing s.d.s are shown.

**Figure 7:**
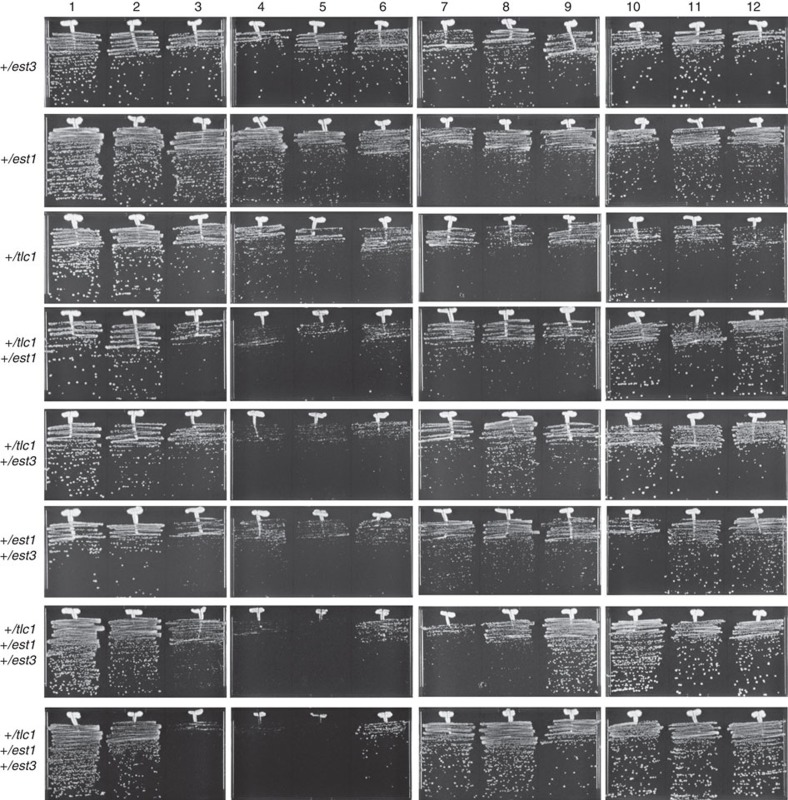
The effect of *TLC1*, *EST1* and *EST3* heterozygosity in 2n−1 (CHRVIII) yeast on cell viability during serial passaging at 38.5 °C. Serial passaging of isogenic aneuploid strains with the relevant genotypes shown on the left.

Consistent with this model, northern blotting analysis revealed a delay in pre-rRNA processing in aneuploids as evident from the accumulation of 35S pre-rRNA (Fig. 5b) and a reduction in the levels of mature 5.8S and 5S RNAs (Fig. 5c). Quantitative mass spectrometry (MS) revealed a ≥ 1.5-fold decrease in the abundance of 39 RPs in aneuploids (Fig. 5d and Supplementary Data Set 1, group 1), accompanied by upregulation of a number of non-RPs (Supplementary Data Set 1, group 2). Furthermore, quantitative western blot analyses showed significantly elevated levels of Est1 and Est3 in aneuploids, particularly at 38.5 °C (Fig. 6). Finally, the defect in rRNA production in aneuploids (Fig. 5b,c) was accompanied by a relative increase in the abundance of the telomerase RNA TLC1 (Fig. 6). This increase in the abundance of the telomerase components was required for suppression of telomerase insufficiency as reductions of Est1, Est3 and TLC1 functions in the aneuploids via introduction of heterozygous deletions in the corresponding genes led to the re-appearance of the viability crisis at 38.5 °C (Fig. 7). We conclude that the aneuploidy-dependent increases in TLC1, Est1 and Est3 partially compensate for the lower Est2 level by promoting telomerase complex formation and/or telomerase recruitment to telomeres, thereby suppressing telomerase insufficiency.

## Discussion

Telomere length equilibrium reflects a balance between telomere shortening due to the end replication problem and telomerase-dependent telomere lengthening. Telomerase insufficiency described here is a situation when telomerase cannot fully compensate for the loss of telomeric repeats during replication and therefore telomeres gradually shorten and a viability crisis due to critically short telomeres occurs. At the growth temperature of 38.5 °C, the levels of the telomerase catalytic subunit Est2 are reduced. Our genetic data suggest that this reduction is responsible for the temperature-induced viability crisis as an additional copy of *EST2* is sufficient to prevent it. Est2 binds stem–loop structures of the telomerase RNA TLC1. It has been shown that Est2 levels are decreased in *tlcΔ* cells^28^ and therefore Est2 binding to TLC1 might be required to stabilize Est2. At the higher growth temperatures, the stability of the duplex regions of TLC1 might be compromised, thereby decreasing the binding of Est2 to the RNA and increasing Est2 instability.

The transition of a haploid yeast population with telomerase insufficiency to aneuploidy is based on a stochastic in nature rather than a developmentally programmed mechanism. Most cells in the senescing population are haploid and eventually die: the very few that survive are likely pre-existent cells, which at some point underwent chromosome mis-segregation and became aneuploid. This aneuploidy, which would confer a growth disadvantage at 30 °C, becomes advantageous at 38.5 °C by allowing cell survival and proliferation as a result of suppression of the temperature-induced telomerase insufficiency. Consequently, the population of survivors seeded by the previously rare aneuploids becomes dominated by aneuploid cells.

The transition of the yeast population from fast-growing haploids to slower-growing near diploids in response to telomerase insufficiency is reversible, as after returning to lower temperatures the aneuploid populations can be outgrown by frequently arising full diploids (Fig. 4b,c). Although these diploids have a haploid-like mating type they could easily mate to similar diploids of the opposite mating type to generate *MATa/a/α/α* quadruploids, which would then generate *MATa/α* diploids during the initial sporulation, and haploids in the following round of sporulation^29^. Therefore, the switch to CHRVIII aneuploidy due to telomerase insufficiency is fully reversible and represents a great example of genome dynamics and plasticity in response to stress to promote cell survival and proliferation.

In the recent years, aneuploidy has been reported as a stress adaptation mechanism in yeast and mammalian cells (reviewed by Chen *et al.*^30^). Aneuploidy is normally not optimal for cell fitness and leads to altered cell physiology and further genomic instability^31^,^32^,^33^. However, aneuploidy can confer growth advantages under stress conditions such as exposure to toxins, high osmolarity or sub-optimal nutrition, and is proposed to drive adaptation of cell populations to changing environment^34^. The previously reported cases of aneuploidy in *S. cerevisiae* involved haploid yeast disomic for one or a few chromosomes. The mechanistic links between gaining an extra chromosome and the resultant phenotypes were often tracked to a gene(s) on that chromosome, which, due to the increase in gene dosage, was expressed at a higher level in the aneuploids, thereby providing growth advantages in the altered environmental conditions. In contrast, our study on yeast adaptation to telomerase insufficiency uncovered a karyotype change from a haploid set of chromosomes to a near-diploid set, involving a monosomy for a single chromosome, CHRVIII. Such a karyotype switch provides the opposite gene dosage adjustment—a twofold decrease in the copy number for the genes on the affected chromosome.

Interestingly, the proteome changes in the CHRVIII aneuploids went far beyond the factors encoded by CHRVIII. We observed lowered abundance of a large pool of RPs most of which were encoded by genes on chromosomes other than CHRVIII. The decreased abundance of RPs could be a result of either lowered protein synthesis or increased protein degradation due to the synthesized RPs not being incorporated into newly made ribosomes due to unbalanced production of RPs^35^. We believe the downregulated synthesis is a more likely scenario since the genetic pursuit of CHRVIII genes relevant to the suppression of telomerase insufficiency identified multiple key players in ribosome biogenesis, such as Kog1, Sch9, Ppr8 and Utp9. The location of the four corresponding genes (and perhaps some other relevant, yet unidentified factors) on CHRVIII makes this chromosome unique for its role in the modulation of ribosome biogenesis via aneuploidy: combined haploinsufficiencies of these factors in CHRVIII aneuploids result in very specific changes in cellular metabolism, where ribosome production rate might be decreased in an energy-saving manner, via decreased synthesis rather than increased degradation of ribosomal components.

Because RPs constitute 80–85% of total cell protein, a 1.5-fold decrease in the abundance of RPs would lead to an approximately threefold increase in non-RPs (the ratio of RPs/non-RPs in euploid cells 80–85% versus 15–20% would become 53–57% versus 43–47% in the aneuploids). Such increases were easily detectable for some of the most abundant non-RPs in the aneuploids in the comparative MS analyses (Supplementary Data Set 1 and Supplementary Tables 1–3). We also detected more than twofold increases in the levels of the telomerase subunits Est1 and Est3, but not Est2, by using quantitative western blotting. Ribosomal RNA constitutes ∼50% of total cellular RNA. On the basis of the observed decreases in the 5S RNA/tRNA and 5.8S RNA/tRNA ratios and the slower processing of 35S pre-rRNA in the CHRVIII aneuploids, an overall increase in non-ribosomal RNA in the aneuploids is highly likely, perhaps due to the haploinsufficiency of *UTP9*. Consistent with this hypothesis, the CHRVIII aneuploidy was also accompanied by a fourfold increase in the telomerase RNA TLC1. Although the abundance of Est2 was unchanged in the aneuploids, the increases in Est1, Est3 and TLC1 levels were necessary for the aneuploids to survive at higher growth temperature (Fig. 7). The increased abundance of the three telomerase components might promote assembly of the fully functional telomerase complex and its recruitment to telomeres, thereby maximizing the usage of the existing Est2 to ensure telomere maintenance and avoiding telomerase insufficiency. Because CHRVIII aneuploidy affects abundance of a variety of other non-RPs, cellular mechanisms and pathways other than telomere maintenance might be affected in a similar manner. Therefore, aneuploidy-dependent modulation of ribosome biogenesis may have evolved as a general mechanism that relies on genome plasticity to re-equilibrate cellular metabolism in response to changing environment by providing compensatory adjustments against growth-limiting factors to maximize cell proliferation.

Interestingly, the abundance of the telomerase catalytic subunit Est2 was not affected by aneuploidy. This suggests that there might be a feedback loop mechanism regulating the amount of Est2 in the cell, or that Est2 synthesis might be co-regulated with ribosome biogenesis. Ribosome biogenesis is modulated in response to growth factors through the TOR pathway. The TOR pathway has been linked to lifespan regulation in single- and multicellular eukaryotes^36^,^37^ as well as to telomere length changes in yeast^38^. Two of the four genes on CHRVIII—*KOG1* and *SCH9*—found to be relevant to the suppression of telomerase insufficiency and code for components of the TORC1 pathway in *S. cerevisiae*. However, the combined haploinsufficiencies of *KOG1* and *SCH9* are not enough to bypass telomerase insufficiency (Fig. 5a). Additional inputs from the insufficiencies in *PRP8*, *UTP9* and other unidentified factors on CHRVIII (since the combination of the four haploinsufficiencies has not completely stabilised CHRVIII) are important for the suppression of telomerase insufficiency. The connection between the TOR pathway, telomere length regulation and lifespan remains very intriguing. Human telomerase has been found in a complex with mTOR^39^ and this association may be part of a regulatory mechanism coordinating telomerase with cell growth and proliferation.

The CHRVIII aneuploids are near diploids that originated from haploid yeast with critically short telomeres. A switch to tetraploidy in response to problem telomeres in precancerous cells has recently been proposed as an early step in cancer development^40^,^41^. Mammalian tetraploid cells are highly unstable and become aneuploid, thereby promoting tumorigenesis^42^. One can easily envision how the selection for aneuploids with upregulation of telomerase could be the driving force for cancer progression at the next stage. Another interesting consideration is whether the well-established connection between ribosomopathies (a group of pathologies linked to ribosome biogenesis) and increased risk of cancer^43^ involves telomerase upregulation. Therefore, studying aneuploidy as a means to regulate telomerase levels may not only further our understanding of how simple organisms adapt to environmental conditions but could also help to elucidate the mechanisms of cancer and telomere syndromes in humans.

## Methods

### Strains and plasmids

Strain genotypes and construction are described in Supplementary Data Set 2. Plasmid pYT72 contains the last 500 bp of *CDC13*-coding sequence fused in frame with the *EST1*-coding sequence followed by 500 bp of *CDC13* 3′ untranslated region, cloned as a HindIII–BamHI fragment in the pRS406 integration vector. When linearized with EcoNI, the plasmid integrates into the endogenous *CDC13* locus thereby fusing the endogenous *CDC13* to *EST1*. pYT100 contains the C-terminal part of *CDC13* fused to *EST2*, in the integration vector pRS406. The plasmid was made by sub-cloning EagI–SalI fragment from pVL1107 (ref. 24) into pRS406. When pYT100 is linearized with NruI, the plasmid integrates at the endogenous *CDC13* locus, thereby fusing the endogenous *CDC13* to *EST2*. The 5- fluoroorotic acid was used to select for cells after spontaneous loss of the fusion construct (after telomere elongation).

To generate a 2n−1 (CHRVIII) aneuploid at 30 °C, *URA3* was integrated near CHRVIII centromere (between *QCR10* and *LEU5*) in a haploid strain with both arms of CHRVIII marked with genetic markers (*NAT* and *HYG*). This haploid was then crossed to another haploid with an unmarked CHRVIII to generate a diploid. The diploid strain was then transformed with a recombinant PCR product, which after recombining into the CHRVIII induces deletion of both *URA3* and the adjacent centromere, thereby leading to CHRVIII loss. Transformants were selected on 5- fluoroorotic acid, screened for the loss of the *NAT* and *HYG* markers and then the CHRVIII loss was confirmed by quantitative pulsed-field gel electrophoresis.

### Yeast passaging and chromosome stability assays

Multiple independent clones were streaked on YPD agar supplemented with additional 0.01% L-tryptophan, 0.01% adenine and 0.003% uracil and incubated at the corresponding temperature for either 2–2.5 days (if grown at 26–37 °C) or 3 days (if grown at 23 or 38.5 °C) before being passaged onto plates with fresh media. To assay CHRVIII stability in diploid cells, both copies of the chromosome were double marked. One copy of the chromosome contained an *HPH* cassette between *SPB1* and *RPL8A* on the right arm and *ARG4* on the left arm. The other copy of CHRVIII had a *NAT* cassette and *arg4::TRP1* at the respective loci. When effects of different deletions on CHRVIII were analysed, such marking was not necessary if a deletion included at least one essential gene. In this case, only the copy with the deletion could be lost and such events were detected by the loss of the marker used to make the deletion. After each passage, cells were tested for the presence of the genetic markers on CHRVIII to calculate CHRVIII stability. Chromosome III stability in *matΔ::KAN /MATα* cells was monitored by the presence of kanamycin resistance and Mat-α.

### FACS analysis

Cells exponentially growing in YPD broth were collected and re-suspended in 70% ethanol. To fix cells, samples were incubated for at least 2 h at room temperature on a rotator, then washed three times with 50 mM sodium citrate and treated with 0.5 mg ml^−1^ RNase A at 37 °C overnight. Cells were washed from RNase A and stained with 1 μM SYBR Green I (Invitrogen) for 1 h at room temperature, sonicated for 4 s and processed immediately using a Becton Dickenson FACS Analyser. The FACS profiles of the experimental samples were compared with the control samples of haploid and diploid strains.

### Telomere analysis by Southern blotting

Telomere length and structure in *S.cerevisiae* were analysed by telomere-specific Southern blotting. Briefly, purified yeast genomic DNA was digested either with KpnI (to analyse Y′ telomere length) or with XhoI (to analyse all telomeres) and resolved on 0.85% agarose gel and blotted onto a charged nylon membrane. Blots were hybridized to either KL1 probe specific to the subtelomeric Y′ repeat^44^, or to (cccaca)_4_ oligonucleotide recognizing telomeric sequences. For *S. pombe* telomere analysis, total genomic DNA was digested with EcoRI and resolved on 1% agarose gel. A TAS1-specific probe^45^ was used for hybridization. To analyse telomeres in *C. vulgaris*, BamHI was used to digest genomic DNA, which then was resolved on 1.2% agarose gel. P-32 end-labelled oligonucleotide ccctaaaccctaaaccctaaaccc was used for Southern blot hybridization.

### Protein analysis by western blotting

For protein analysis, 5–10 optical densities (ODs; OD_600_) of cells actively dividing either on plates (for Rad53-HA) or in liquid culture (13myc-tagged Est1, Est2 or Est3) were used to prepare total protein extracts by trichloroacetic acid precipitation^46^. Protein concentration in each sample was measured using Bradford reagent (Bio-Rad) and equal amount of protein was loaded in each lane on SDS–polyacrylamide gel electrophoresis. The resolved proteins were transferred onto a polyvinylidene difluoride membrane and incubated with a corresponding primary antibody: rat monoclonal anti-HA at 1: 1,000 dilution (clone 3F10, Covance) or mouse monoclonal anti-myc at 1:1,000 dilution (9E10, Covance). Horseradish peroxidase -conjugated goat anti-rat antibody at 1:10,000 dilution and ECL Plus detection kit (GE Healthcare) were then used to visualize Rad53-HA. For the quantitative analysis of the myc-tagged Est1, Est2 and Est3, secondary antibodies (goat anti-mouse IgG at 1:25,000) covalently linked to infrared fluorophore Alexa Fluor 680 (Invitrogen) were used. The blots were then scanned using an Odyssey infrared imager and analysed with Image Studio 2.0 software (LI-Cor Biosciences). The relative amounts of the telomerase components were expressed as a myc signal normalized against total protein. Three biological repeats using at least two independently constructed strains were used for each measurement to calculate averages and s.d.s. Uncropped blots of all gels appear as Supplementary Fig. 9.

### RNA isolation and analysis

For total RNA isolation, yeast cultures were grown to mid-exponential phase at 30 or 38.5 °C and 15 ODs of each culture were collected by centrifugation. Total RNA from each sample was isolated using the hot phenol method^47^.

To quantify the relative amount of TLC1 RNA in cells with different genotypes, 5 μg of total RNA was glyoxyl denatured and resolved on 1.2% agarose gel^48^. The gel was stained with ethidium bromide and imaged using Bio-Rad gel documentation system. The relative amount of the 18S RNA in each lane was quantified using Image Lab software (Bio-Rad). RNA was blotted onto a positively charged nylon membrane and hybridized with a ^32^P-labelled probe (Prime-It II kit for random prime labelling, Agilent) corresponding to the BglII–BclI fragment of the *TLC1* gene. The blots were exposed to phosphorstorage screens and scanned using Typhoon scanner. The TLC1 signal in each lane was quantified using ImageQuant software (GE Healthcare). For comparative analysis, TLC1 signal was normalized against 18S RNA signal. Three biological repeats were used for each measurement to calculate averages and s.d.s.

For quantitative analysis of rRNA, 15 μg of total RNA of each sample was mixed with formamide loading buffer, heated for 5 min at 75 °C and loaded on a denaturing gel: 8% acrylamide (19:1 acrylamide to bis-acrylamide ratio) 7.5 M urea gel in TBE buffer. The gel was run until bromophenol blue was released into the lower chamber. The RNA was stained with ethidium bromide imaged using Bio-Rad gel documentation system. The relative amount of the 5.8S, 5S and tRNA in each lane was quantified using Image Lab software (Bio-Rad). Three biological repeats were used for each measurement to calculate averages and s.d.s.

To analyse the pre-rRNA processing, 5 μg of total RNA was glyoxyl denatured and resolved on 1.2% agarose gel^48^. The gel was stained with ethidium bromide and imaged using Bio-Rad gel documentation system. RNA was transferred onto a positively charged nylon membrane and hybridized with ^32^P-labelled oligonucleotides 5′-TGTTACCTCTGGGCCC-3′ and 5′-CGGTTTTAATTGTCCTA-3′ to visualize 35S–27S rRNA and 20S rRNA, respectively.

### Array comparative genome hybridization

Total genomic DNA was purified from telomerase insufficiency survivors and their derivatives as well as from a diploid control strain NK2442 which was used as a reference. DNA samples were submitted to Nimblegen (Roche) for labelling and hybridization to custom designed 135K arrays tiling 16 chromosomes (non-repetitive sequences), mitochondrion and Y′ repeats of *S. cerevisiae*.

### Analysis of relative protein abundance by MS

Three independently generated 2n−1 aneuploids (genetically engineered at 30 °C as described above) along with their parental diploid strain were used in the experiment. Cells were grown to mid-logarithmic stage at 38.5 °C in rich media, collected by centrifugation and frozen at −80 °C.

The trichloroacetic acid–acetone-precipitated pellets of total yeast protein samples^46^ were reconstituted in 250 μl of 8 M urea, and protein concentration was measured using Bradford reagent (Bio-Rad). One milligram of protein extract was mixed with 25 μl of 1 M ammonium bicarbonate and 25 μl of 200 mM dithiothreitol to enable denaturation and reduction of the proteins during incubation at room temperature for 30 min. For cysteine alkylation, 25 μl of 500 mM iodoacetamide was added to each sample and incubated for 1 h at room temperature. Samples were diluted with water to reduce urea concentration to 2 M, 20 μg of trypsin was added to each sample and the protein digestions were performed overnight at room temperature. Peptide extracts were then cleaned on Bond Elut 25 mg SPE reverse phase according to supplier’s recommendations (Agilent, UK), then dried in a speedvac concentrator (ThermoFisher, UK) and stored at −20 °C. The dried peptide samples were re-suspended in 0.05% trifluoroacetic acid (TFA) to a final concentration of 1 μg μl^−1^. These samples were filtered using Millex filter (Millipore, UK) before being subjected to HPLC–MS analysis (5 μg of each sample was injected).

Nano-HPLC-MS/MS analysis was performed using an online system consisting of a nano-pump (Dionex Ultimate 3000, Thermo-Fisher, UK) coupled to a QExactive instrument (Thermo-Fisher, UK) with a pre-column of 300 μm × 5 mm (Acclaim Pepmap, 5 μm particle size) connected to a column of 75 μm × 50 cm (Acclaim Pepmap, 3 μm particle size). Samples were analysed on a 2-h gradient in data-dependent analysis (1 survey scan at 70 k resolution followed by the top 10 MS/MS at 17.5 k resolution).

Progenesis (version 4.1 Nonlinear Dynamics, UK) was used for LC–MS label-free quantitation. Only MS/MS peaks with a charge of 2+, 3+ or 4+ were taken into account for the total number of ‘Feature’ (signal at one particular retention time and *m*/*z*) and only the five most intense spectra per ‘Feature’ were considered in the analysis. Normalization was first performed based on the sum of the ion intensities of these sets of multi-charged ions (2+, 3+ and 4+). The associated unique peptide ion intensities for a specific protein were then summed to generate an abundance value. The abundance was then transformed using an ArcSinH function (a log transform is not ideal considering the significant amount of near-zero measurements generated by the current method of detection). On the basis of the abundance values calculated in three biological repeats for both diploid and aneuploids stains, within group means were calculated and from there the fold changes (in comparison with wild-type) were evaluated. One-way analysis of variance was used to calculate the *P* values based on the transformed values. Differentially expressed proteins were only considered significant in current study if the following conditions were fulfilled: (i) *P* values (pair wise) <0.05; (ii) number of peptides detected and used in quantification per protein was equal to or more than 2; and (iii) absolute fold change was at least 1.5.

Data from MS/MS spectra were searched using MASCOT Versions 2.4 (Matrix Science Ltd, UK) against the *S. cerevisiae* subset of the NCBI protein database including contaminant and reverse sequence for a total of 12,288 sequences with maximum missed-cut value set to 2. Following features were used in all searches: (i) variable methionine oxidation; (ii) fixed cysteine carbamidomethylation; (iii) precursor mass tolerance of 10 p.p.m.; (iv) MS/MS tolerance of 0.05 AMU; (v) significance threshold (*p*) below 0.05 (MudPIT scoring); and (vi) final peptide score of 20, which correspond to <1% FDR. Gene name and secondary Identifier (with gene locus information) was performed using Saccharomyces Genome Database (October 2014, www.yeastgenome.org). GO enrichment strategy was performed on the two significantly differentially expressed group (up- and downregulated) using a two unranked lists of gene approach (each significantly differentially expressed groups against the pool of identified protein, which are not differentially expressed identified in the study) using GOrilla tools^49^.

## Additional information

**How to cite this article:** Millet, C. *et al.* Cell populations can use aneuploidy to survive telomerase insufficiency. *Nat. Commun.* 6:8664 doi: 10.1038/ncomms9664 (2015).

## Supplementary Material

Supplementary Information Supplementary Figures 1-8 and Supplementary Tables 1-3.

Supplementary Data 1 Relative abundance of proteins in diploid and chromosome VIII monosomic yeast analysed by LC-MS.

Supplementary Data 2 Strains of S. cerevisiae A364a used in the study.
