# Supplementary material for: Cell populations can use aneuploidy to survive telomerase insufficiency
Source: Nat Commun. 2015 Oct 22;6:8664. doi: 10.1038/ncomms9664 (PMC4627575; doi:10.1038/ncomms9664)
Supplement: Supplementary Information — Supplementary Figures 1-8 and Supplementary Tables 1-3. [file ncomms9664-s1.pdf]

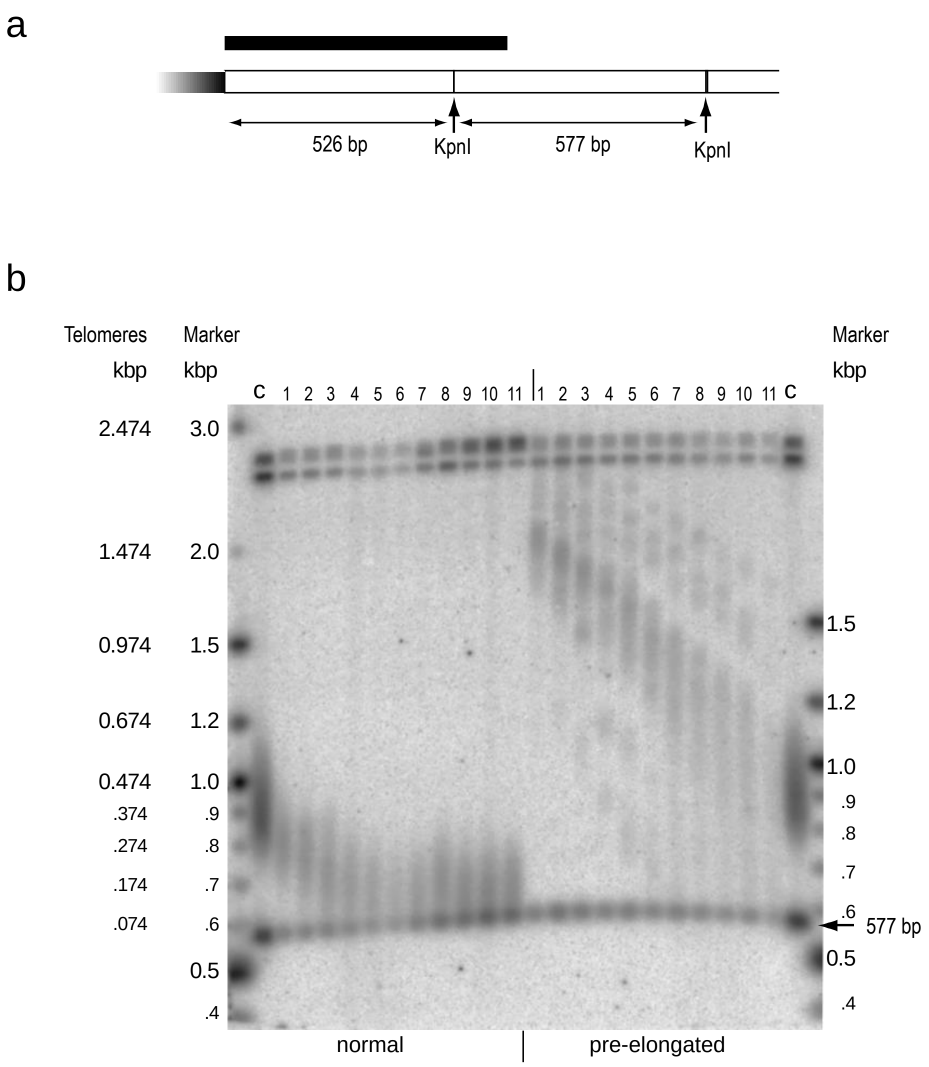

Supplementary Figure 1. Telomere length analysis in yeast undergoing cellular senescence at 38.5°C. a, Schematic of a Y' telomere with the adjacent sub-telomeric region. Telomeric $(TG_{1-3})_n$ repeats are depicted as a grey-scale gradient on the left and the Y' repeat sequence is shown in white. One KpnI restriction site is located 526 bp away from the telomere-Y' junction and another one is 577 bp away from the first one as indicated by vertical arrows. The KL1 probe is shown as a black bar at the top. **b**, Y' telomere length analysis by Southern hybridisation (DNA samples used in Fig. 3c). A control sample (C) from wild type strain grown at 30°C is the lanes labelled with C. Genomic DNA samples were digested with KpnI, resolved on 0.85% agarose gel alongside with a DNA size marker (first and last lanes) and hybridised to the KL1 probe and a marker-specific probe. Because the KL1 probe covers 630 bp of telomere proximal Y' sequence, both the 577 bp KpnI fragment and the terminal restriction fragment (TRF) are detected. Sizes of the marker bands are shown next to the blot image on the left and on the right. The 577 bp KpnI fragment is indicated by the arrow on the right. On the left, Y' telomere length corresponding to a TRF migrating at the position of each marker band is calculated (the length of the marker DNA minus 526 bp of sub-telomeric sequence). Notice that the shortest TRFs in the

samples from passages 5-11 of the “normal” set have the same gel mobility as the 577 bp band. A 577 bp TRF corresponds to 51 bp telomere length (577-526 bp). The average Y’ TRFs in these cells are ~ 650 bp, i.e. the telomeres are ~125 bp long.

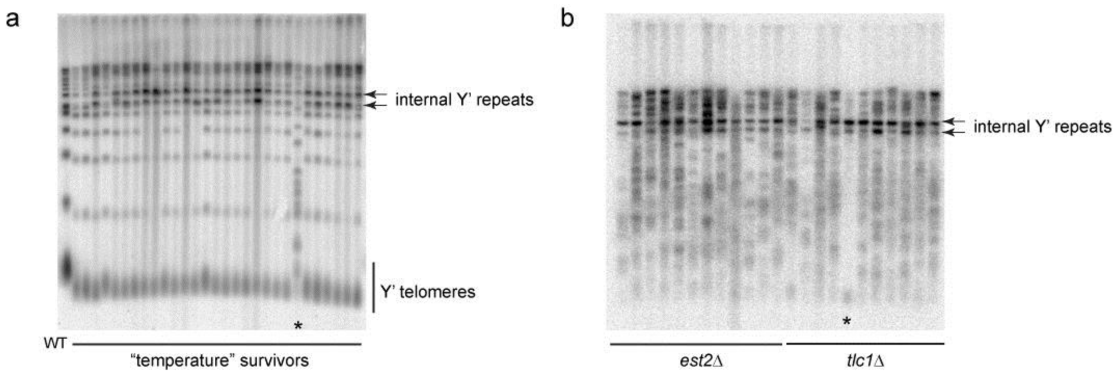

Supplementary Figure 2. "Temperature" survivors maintain short but stable telomeres at 38.5°C. Telomere length analysis by Southern blotting. **a**. Temperature survivors after 12 passages at 38.5°C. A wild type control (WT) with telomeres equilibrated at 30°C is shown in the first lane. Notice, that some survivors showed amplification of internal Y' repeats, characteristic of type I survivors. However, their X-telomeres have not acquired terminal Y' repeats. Among many survivors (over 100), only one (marked with an asterisk) had telomeres somewhat similar to type II survivors but the clone went through a strong viability crisis upon *TLC1* deletion. **b**. telomerase-deficiency at 38.5°C leads to the formation of typical type I (marked with an asterisk) and type II (the rest) survivors.

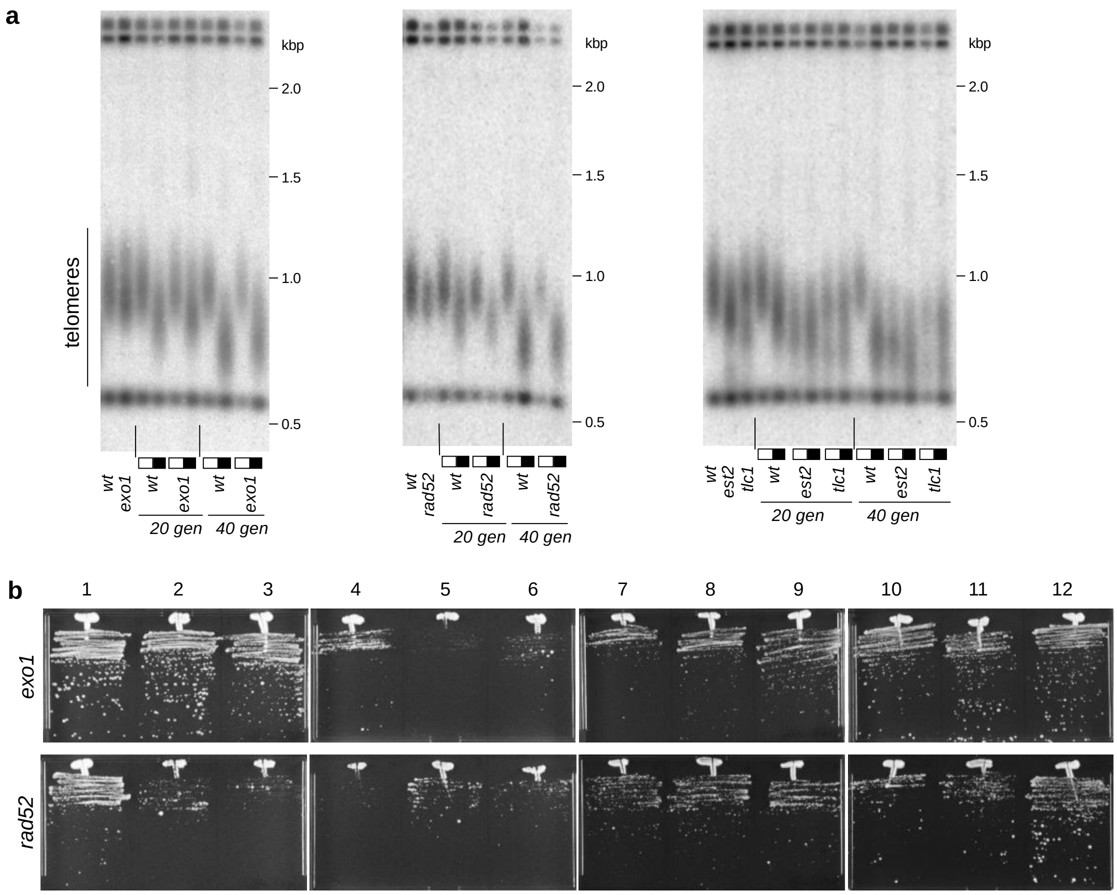

Supplementary Figure 3. Telomere shortening upon telomerase loss is temperature-independent. a, Telomere length analysis by Southern blotting. The first 2 (left and middle panels) or 3 (right panel) lanes show telomeres of wild-type (wt) and a corresponding mutant strains before they were split for passaging at 26°C (white squares) and 38.5°C (black squares) for the number of generation indicated along the bottom. **b**, Serial passaging of *exo1* and *rad52* deletion mutants at 38.5°C.

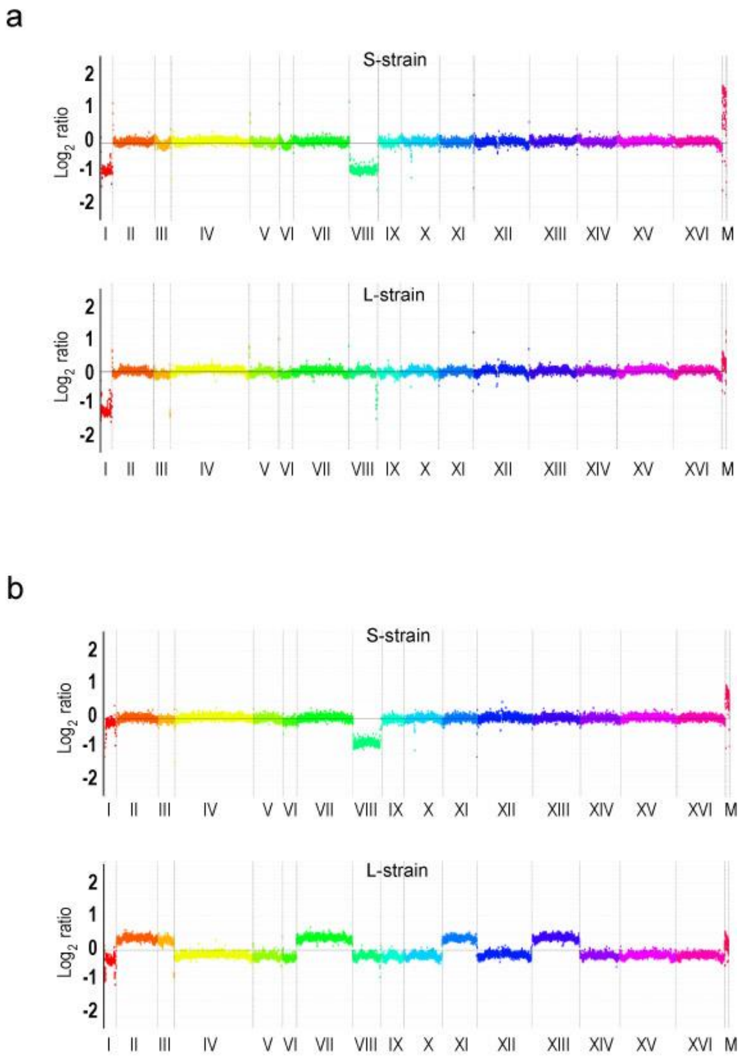

Supplementary Figure 4. aCGH analysis of two dTI-survivors (S-strains) and their L-strain derivatives. Each pair is shown in a separate panel. Notice that the dTI-survivor in panel **a** is aneuploid for both chromosome VIII and chromosome I but its L-strain derivative regained only chromosome VIII. The dTI-survivor in panel **b** is aneuploid for chromosome VIII but regaining the second copy of chromosome VIII was accompanied by acquiring third copies of chromosomes II, III, VII, XI, and XIII.

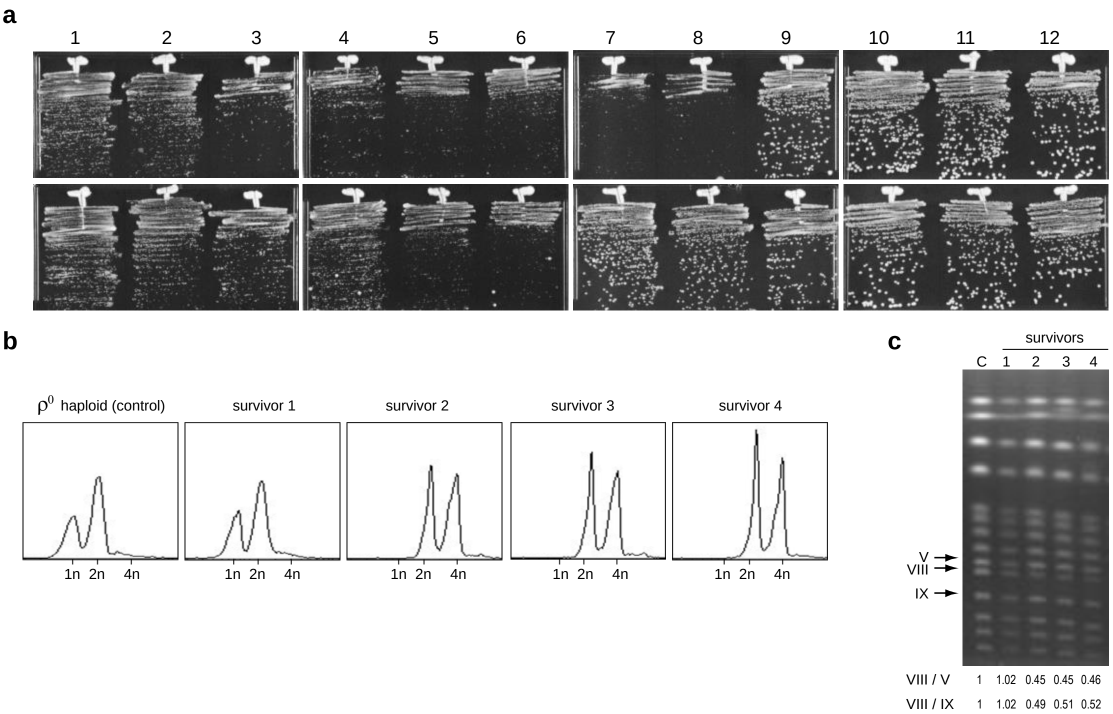

Supplementary Figure 5. Mitochondrial DNA is not required for the formation of aneuploid survivors at 38.5°C. a, Serial passaging of ρ^0 yeast at 38.5°C leads to a viability crisis and formation of survivors with healthy growth. Two independent clones are shown. **b**, FACS profile of four independent ρ^0 survivors along with the parental strain (control). While the survivor 1 is similar to the haploid control the survivors 2-4 have diploid-like FACS profiles. **c**. PFGE was used to analyse the karyotypes of the survivors 1-4 and the control strain (C) from **b** as shown above the gel image. The analysis of the relative amount of CHRVIII normalised against either CHRV or CHRIX (the relevant chromosomes are indicated by arrows on the left) suggests a single copy of CHRVIII in the diploid-like survivors 2-4.

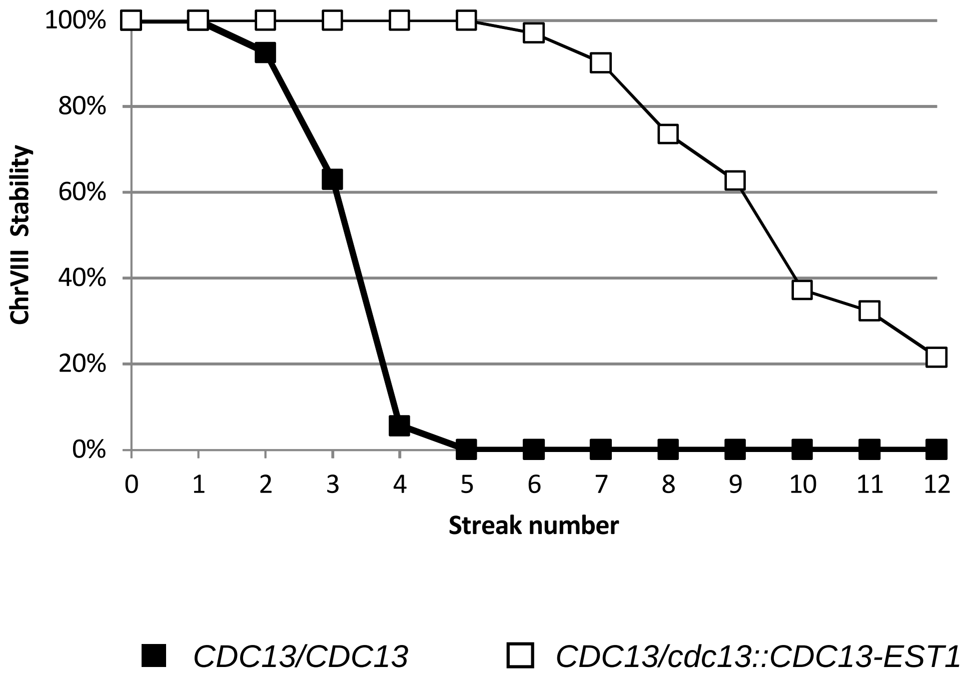

Supplementary Figure 6. Chromosome VIII stability in *matΔ/MATα* diploids and their derivatives with the *CDC13-EST1* gene fusion. Both copies of CHRVIII carried a marker on either arm (*NAT or HYG* at VIIIL and *arg4::TRP1 or ARG4* at VIIIR) to allow monitoring chromosome VIII loss. 54 clones for each genotype were passaged at 38.5 °C and chromosome VIII stability was scored after each passage by the presence of the genetic markers. Simultaneous loss of either Nat^R and Trp^+ or Hyg^R and Arg^+ was scored as a loss of one of the copies of chromosome VIII. The Cdc13-Est1 fusion protein is known to promote telomerase recruitment to telomeres, thereby increasing the telomere length equilibrium.

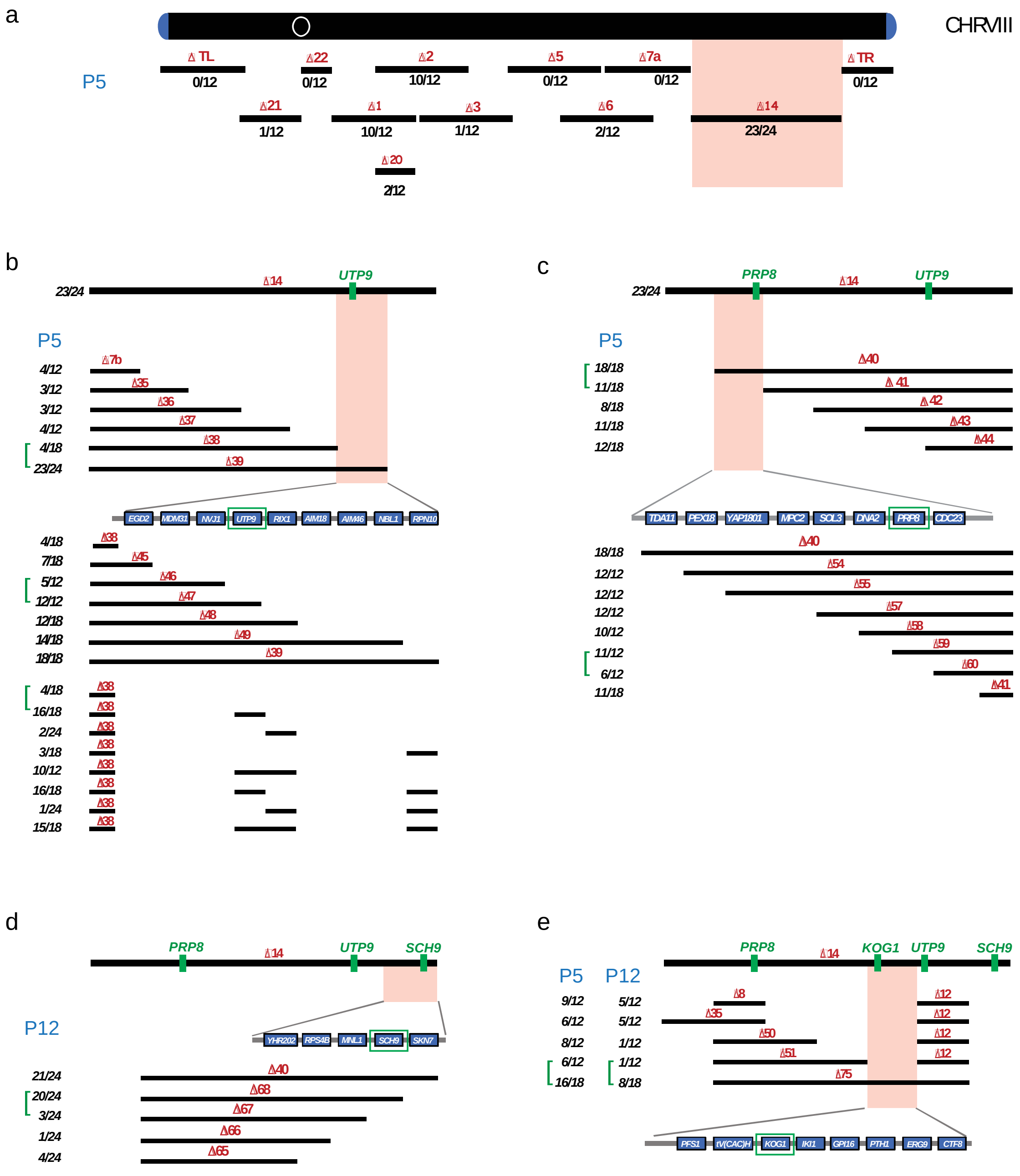

Supplementary Figure 7. Deletion-based screening of chromosome VIII for genes which stabilize the chromosome when present in a single copy in a diploid grown at 38.5°C. (**a-e**) Deletions are shown as black bars spanning the corresponding regions deleted in a heterozygous diploid. The name of each deletion is shown in red above the bar and the spanning region for each deletion can be found in the Supplementary Dataset 2. The stability of CHRVIII with the corresponding deletion is presented as a ratio of colonies retaining two copies of CHRVIII at a given passage (P5 or P12, in blue) to the number of colonies taken into the experiment and shown below each deletion in panel **a** and on the left of the corresponding deletions in panels **b-e**. Green brackets indicate pairs of deletions, comparison of which allowed identification of chromosomal regions (highlighted in pink) or genes (boxed in green rectangles) affecting CHRVIII stability. **a**, Initial analysis of the set of deletions spanning CHRVIII identified deletion 14 (Δ14) as a region stabilizing the

chromosome under the conditions of telomerase insufficiency. Breaking down deletion 14 into a set of smaller non-overlapping deletions led to the disappearance of the stabilizing effect (data not shown) suggesting that more than one gene within the deleted region was involved in the phenotype. Therefore, a strategy of overlapping deletions was used for further analysis (panels **b-e**). **b**, *UTP9* gene copy number is important for CHRVIII stability. First, the region between *EDG2* and *RPN10*, which was a part of the deletion 39 but not deletion 38, was identified. A series of deletions longer than Δ38 but shorter than Δ39 (Δ45-Δ49) were constructed and the difference in CHRVIII stability between Δ46 and Δ47 pointed towards *UTP9*. However, since the CHRVIII stability also varied in the pairs Δ47/Δ48 and Δ49/Δ39 we constructed a series of strains were all possible combinations of *UTP9*, *RIX1*, and *RPN10* gene deletions were added to Δ38 on the same chromosome. The data were consistent with *UTP9* having the major effect on CHRVIII stability. **c**, *PRP8* is the second identified gene with an effect on CHRVIII stability. Similar approach was used as in **b**, except all the overlapping deletions originated on the other side of Δ14 (at *SKN7*). **d**, Identification of *SCH9* as a third gene involved in CHRVIII stability. All the deletions analysed originated from *TDA11* on the left side. They had a strong stabilizing effect at passage 5 and therefore, CHRVIII stability at passage 12 was analysed to reveal the phenotypical differences between the strains. **e**, *KOG1* is another gene-candidate relevant to the CHRVIII stability. Because Δ75 confers a stronger stabilizing effect than the combination of Δ51 and Δ12, the *PFS1-CTF8* region may contain another gene involved in CHRVIII stability. We chose *KOG1* for further analysis (see Fig. 5a) as among all the genes in the region, *KOG1* was the only one similar to *UTP9, PRP8,* and *SCH9* in their common connection to ribosome biogenesis.

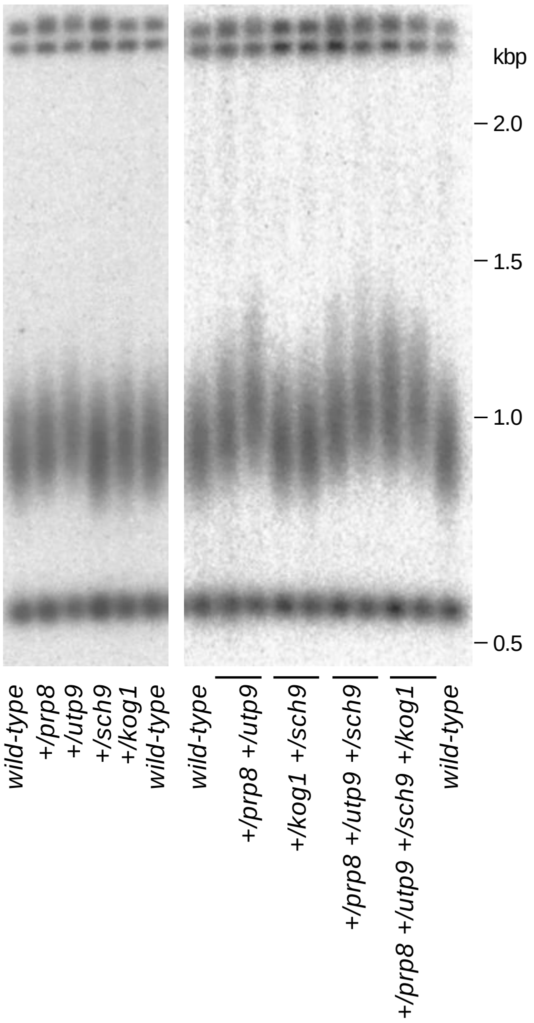

Supplementary Figure 8. Analysis of telomere length at 30°C in the heterozygous diploid mutants with increased CHRVIII stability. Cells were propagated for 80 generations at 30°C and the DNA samples were analysed for telomere length by Southern blotting. Notice that the *UTP9* haploinsufficiency causes slight telomere lengthening which is increased by the *PRP8* haploinsufficiency in the *+/prp8 +/utp9* mutant but not further enhanced by the deletions of *SCH9* in the triple mutant or *KOG1* in the quadruple mutant.

Supplementary Table 1: GO term enrichment associated to the proteins found down regulated in the Aneuploid group (Biological Processes)

GO term	Description	P-value[a]	FDR q-value[b]	Enrichment[c]
GO:0002181	cytoplasmic translation	1.92E-19	5.32E-16	5.1
GO:0006412	Translation	4.25E-15	5.90E-12	3.5
GO:0034645	cellular macromolecule biosynthetic process	5.24E-11	4.85E-08	2.4
GO:0009059	macromolecule biosynthetic process	1.34E-10	9.26E-08	2.4
GO:1901576	organic substance biosynthetic process	3.02E-09	1.67E-06	1.7
GO:0044249	cellular biosynthetic process	3.83E-09	1.77E-06	1.8
GO:0009058	biosynthetic process	5.53E-09	2.19E-06	1.7
GO:0044267	cellular protein metabolic process	1.35E-07	4.69E-05	2.0
GO:0019538	protein metabolic process	1.09E-05	3.36E-03	1.7
GO:0030490	maturation of SSU-rRNA	1.80E-05	4.99E-03	3.9
GO:0008152	metabolic process	7.80E-05	1.97E-02	1.2
GO:0044237	cellular metabolic process	9.42E-05	2.18E-02	1.2
GO:0000462	maturation of SSU-rRNA from tricistronic rRNA transcript	1.68E-04	3.59E-02	3.6
GO:0044260	cellular macromolecule metabolic process	2.45E-04	4.85E-02	1.4

a) s the enrichment p-value computed. This p-value is not corrected for multiple testing.

b) The correction of the above p-value for multiple testing using the Benjamini and Hochberg method.

c) Enrichment = (b/n) / (B/N) where N: total number of genes; B: total number of genes associated with a specific GO term; n: number of genes in the top of the user's input list or in the target set when appropriate; b: number of genes in the intersection

Supplementary Table 2: GO term enrichment associated to the proteins found down regulated in the Aneuploid group (Molecular Functions)

GO term	Description	P-value[a]	FDR q-value[b]	Enrichment[c]
GO:0003735	structural constituent of ribosome	3.12E-21	3.78E-18	5.8
GO:0005198	structural molecule activity	1.40E-16	8.51E-14	4.1
GO:0019843	rRNA binding	2.27E-06	9.17E-04	5.5

a) s the enrichment p-value computed. This p-value is not corrected for multiple testing.

b) The correction of the above p-value for multiple testing using the Benjamini and Hochberg method.

c) Enrichment = (b/n) / (B/N) where N: total number of genes; B: total number of genes associated with a specific GO term; n: number of genes in the top of the user's input list or in the target set when appropriate; b: number of genes in the intersection

Supplementary Table 3: GO term enrichment associated to the proteins found down regulated in the Aneuploid group (Cellular Components)

GO term	Description	P-value[a]	FDR q-value[b]	Enrichment[c]
GO:0044391	ribosomal subunit	1.09E-19	6.03E-17	5.2
GO:0005622	Intracellular	1.37E-17	3.79E-15	5.1
GO:0005840	Ribosome	6.70E-16	1.24E-13	3.9
GO:0044445	cytosolic part	8.40E-14	1.16E-11	3.5
GO:0022627	cytosolic small ribosomal subunit	2.98E-11	3.30E-09	5.8
GO:0015935	small ribosomal subunit	5.69E-11	5.25E-09	5.6
GO:0030529	ribonucleoprotein complex	1.10E-10	8.72E-09	2.3
GO:0022625	cytosolic large ribosomal subunit	1.19E-09	8.24E-08	5.2
GO:0015934	large ribosomal subunit	5.61E-09	3.45E-07	4.8
GO:0043228	non-membrane-bounded organelle	6.40E-06	3.54E-04	1.7
GO:0043232	intracellular non-membrane-bounded organelle	6.40E-06	3.22E-04	1.7
GO:0030686	90S preribosome	1.89E-04	8.71E-03	3.3
GO:0030684	preribosome	4.84E-04	2.06E-02	2.4

a) s the enrichment p-value computed. This p-value is not corrected for multiple testing.

b) The correction of the above p-value for multiple testing using the Benjamini and Hochberg method.

c) Enrichment = (b/n) / (B/N) where N: total number of genes; B: total number of genes associated with a specific GO term; n: number of genes in the top of the user's input list or in the target set when appropriate; b: number of genes in the intersection.
